# Transcriptomic analysis of transformed small-cell lung cancer from *EGFR*-mutated lung adenocarcinoma reveals distinct subgroups and precision therapy opportunities

**DOI:** 10.1186/s40364-025-00789-9

**Published:** 2025-05-28

**Authors:** Hao Sun, Chan-Yuan Zhang, Xiu-Hao Zhang, Zai-Xian Tai, Jun-Wei Su, Xiao-Cheng Lin, Shi-Ling Zhang, Yu-Fa Li, Chao Zhang, Miao Cai, Xu-Chao Zhang, Hua-Jun Chen, Qing Zhou, Yi-Long Wu, Wei-Neng Feng, Jin-Ji Yang

**Affiliations:** 1 Guangdong Lung Cancer Institute, Guangdong Provincial People’s Hospital (Guangdong Academy of Medical Sciences), Southern Medical University, No 106, Zhongshan Second Road, Guangzhou, 510080 China; 2 Department of Pulmonary Oncology, The First People’s Hospital of Foshan, Foshan, Guangdong 528000 China; 3 Geneplus-Shenzhen, Shenzhen, China; 4 Geneplus-Beijing, Beijing, China

**Keywords:** *EGFR*-mutant, Non-small cell lung cancer, Small-cell lung cancer, Transformation, Molecular subtypes

## Abstract

**Background:**

Small-cell lung cancer (SCLC) transformation is one of the major mechanisms of resistance to Epidermal Growth Factor Receptor tyrosine kinase inhibitors (EGFR-TKIs). Chemotherapy is typically the recommended treatment for transformed SCLC (T-SCLC), similar to primary SCLC. However, the benefits of chemotherapy alone are minimal. Prior research highlights differences between the biological traits of T-SCLC and primary SCLC or *EGFR*-mutated lung adenocarcinoma (LUAD). This study aims to elucidate the molecular characteristics of T-SCLC and identify potential treatment modalities.

**Methods:**

We retrospectively collected tissue samples from LUAD, T-SCLC post-EGFR-TKI resistance, and primary SCLC. Genomics, transcriptomics, and proteomics were performed to clarify the differences between T-SCLC, LUAD, and primary SCLC. Hierarchical clustering analysis was then used to categorize the molecular subtype of T-SCLC, followed by a survival analysis based on these subtypes.

**Results:**

A study involving 61 patients investigated differences between LUAD, SCLC, and primary SCLC. RNA sequencing revealed distinct gene expression variations, particularly up-regulation in *PPM1E*, *INSM1*, and *KCNC1* genes in T-SCLC. Pathway analysis linked T-SCLC to the cell cycle and neural differentiation. By conducting Hierarchical clustering analysis on RNA-seq data, the entire population can be categorized into two distinct groups. While certain T-SCLC showed similarities and differences compared to SCLC, with subtypes: LUAD-like and Non-LUAD-like. Notably, the LUAD-like subtype had significantly higher NKX2-1 expression (mean 371.8 vs. 41.8, *P* < 0.0001). T-SCLC treatment approaches were categorized into matched and unmatched groups, delineated by the alignment of specific therapies with corresponding pathologies. The matched group (13 cases) exhibited a significantly prolonged median progression-free survival compared to the unmatched group (10 cases) (5.4 months vs. 3.6 months, *P* = 0.02).

**Conclusions:**

T-SCLC exhibits marked molecular distinctiveness, setting it apart not only from LUAD but also from classical SCLC. This distinction extends to its classification into two discernible molecular subtypes: LUAD-like and Non-LUAD-like. Customizing therapeutic protocols to align with these specific subtypes have the potential to identify the most appropriate treatment for T-SCLC.

**Graphical Abstract:**

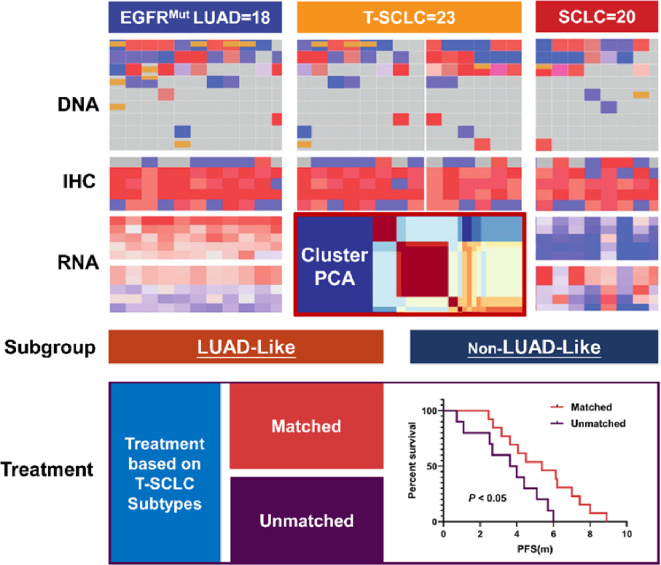

**Supplementary Information:**

The online version contains supplementary material available at 10.1186/s40364-025-00789-9.

## Introduction

Epidermal Growth Factor Receptor (EGFR) tyrosine kinase inhibitors (TKIs) have demonstrated dramatic efficacy for *EGFR*-mutated non-small cell lung cancer (NSCLC), however, acquired drug resistance inevitably occurs at a median time of approximately 12 to 20 months during targeted therapy [1–4]. Small-cell lung cancer (SCLC) transformation, as one of the major mechanisms of EGFR-TKI resistance, was reported in 3-14% [5–7]. SCLC transformation is one of the common manifestations of lineage plasticity, and it is the process by which lung adenocarcinoma cells acquire the phenotypic characteristics of SCLC lineage. However, the specific mechanism of transformation remains unclear. In particular, the inactivation of RB1 and TP53 promotes SCLC transformation by lung adenocarcinoma [8, 9].

However, less is known about the biological characteristics of Transformed-SCLC (T-SCLC). Previous in vitro tests have indicated that T-SCLC typically expresses neuroendocrine (NE) markers, which are similar to classic SCLC in RNA profiles [10]. Platinum-based chemotherapy as first-line treatment for de novo SCLC has demonstrated high response rates with objective response rates of approximately 45–70% [11–13]. These findings support the adoption of the standard etoposide/platinum (EP) chemotherapy regimen in T-SCLC treatment, based on its established efficacy in de novo SCLC [14]. Although 54% of patients had a clinical response to EP regimen in previous retrospective studies, the median progression-free survival (mPFS) was only 3.4 months [15]. There is currently no universally accepted standard treatment for T-SCLC.

Patients with *EGFR* mutations were more prone to SCLC transformation than patients with wild-type *EGFR* mutations [16]. EGFR-TKI combined with chemotherapy may be a potential treatment option for T-SCLC, and previous studies have also confirmed that combined therapy can significantly prolong PFS [17, 18]. Nevertheless, not all patients benefit equally from combined EGFR-TKI treatment, so identifying the target population is extremely valuable.

In the era of precision medicine, this classification has certain limitations based on pathology to formulate treatment options. There has been little progress in the medical treatment of SCLC considered homogeneous disease, even though receiving immunotherapy extended the overall survival of these patients by 2–5 months [11, 19, 20]. Recent studies have demonstrated that SCLC subtypes were characterized by 4 major transcription factors, ASCL1, NEUROD1, and POU2F3 [21, 22]. Molecular typing has important guiding significance for treatment selection and prognosis. Similarly, previous explorations in characterizing the heterogeneity of T-SCLC through immunohistochemistry and transcriptome sequencing provided initial insights [23, 24]. It is gradually recognized that tumour heterogeneity also exists in T-SCLC, but it is unclear whether heterogeneity affects clinical decision making. Until now, no effective molecular markers have been found to tailor the individualized treatment programs for T-SCLC.

T-SCLC is a highly aggressive disease and is usually associated with poor clinical outcomes. It is important to clarify the molecular characteristics of transformed-SCLC (T-SCLC) and explore the appropriate treatment modality for it. Therefore, in this study, we performed multi-omics analyses of the genomics, transcriptomics, and proteomics to compare the differences among T-SCLC, lung adenocarcinoma (LUAD) and SCLC. Our strategy provides new insights into the molecular subtyping of T-SCLC and provides a basis for subsequent treatment selection.

## Methods

### Patients and data collection

Patients with SCLC transformed from *EGFR*-mutated adenocarcinoma were screened after acquired resistance to EGFR-TKIs in Guangdong Provincial People’s Hospital Lung Cancer Database from October 30, 2018, to September 30, 2021. All the patients were involved according to the following criteria: baseline pathological diagnosis of adenocarcinoma with *EGFR* mutation; received EGFR-TKI therapy and acquired drug resistance; re-biopsy confirmed SCLC; sufficient samples to satisfy testing. Patients initially diagnosed with mixed pathology were excluded. As a comparison, we also included untransformed TKI-resistant adenocarcinoma and de novo advanced SCLC samples from the same period.

Detailed data collection on demographic information, histopathology, molecular pathology, treatment regimens, and survival outcomes were recorded. Ethics approval was approved by the Research Ethics Committee of the Guangdong Provincial People’s Hospital, Guangdong Academy of Medical Sciences (Guangzhou, China).

### Pathological diagnosis and immunohistochemistry (IHC)

The obtained lung cancer tissue samples were fixed in 4% formalin solution and embedded in paraffin wax blocks. The prepared wax blocks were subjected to hematoxylin and eosin (H&E) and immunohistochemical staining respectively. The included patients were initially pathologically diagnosed as adenocarcinoma, and the specific antibodies CK7, TTF-1 and NapsinA were positive. Patients undergoing SCLC transformation were eventually confirmed by histopathology. Synaptophysin (Syn), Chromogranin A (CgA), and CD56 were specific clinical diagnosis indexes for T-SCLC. Similarly, adenocarcinoma and de novo SCLC were diagnosed using corresponding antibodies, including TTF-1, CK7, NapsinA, Syn, CgA, and CD56. Evaluation of IHC staining results was performed by two independent pathologists. Positive cells were scored as expressing complete absence of staining or less than 10% (−), 10–25% (+), 25–75% (++), and more than 75% strong positive (+++).

### Sample collection

Tumour tissues or metastatic tumour tissues were collected by tissue biopsy and prepared into fresh-frozen sample at -80 °C or formalin-fixed paraffin-embedded (FFPE) samples. Total RNA extraction and genomic DNA were performed with RNAprep Pure FFPE Kit (Tiangen Biotech, Beijing, China) and TIANamp Genomic DNA kit (Tiangen Biotech, Beijing, China) following the manufacturer’s instructions.

### Next-generation sequencing (NGS) and RNA sequencing

For the identification of genomic alterations, the vast majority of biological samples were subjected to NGS, and a few were an amplification refractory mutation system (ARMS). Tumour samples from enrolled patients were submitted to three commercial laboratories for genetic analysis. Targeted-capture sequencing was performed using mature commercial panels containing at least 139 lung cancer-associated variant genes or up to 1021 gene alterations. Captured libraries were then pair-end sequenced with Illumina sequencing platform (Illumina, CA, USA) or Geneplus sequencing platform (Geneplus, Beijing, China) following the manufacturer’s guidance.

Total RNA was digested with TIANSeq rRNA Depletion Kit (Tiangen Biotech, Beijing, China) and the quality of purified RNA was determined by Agilent 2100 bioanalyzer (Agilent, WA, USA). Constructed library was then pair-end sequenced in 100-bp lengths with Geneplus-2000 sequencing platform (Geneplus, Beijing, China) following the manufacturer’s guidance. Raw data from next-generation sequencing was then filtered to remove low-quality reads and adaptor sequence. Reads were further aligned to the reference human genome (hg19) using STAR software [25].

### Bioinformatic analysis

The single nucleotide variation (SNV) and copy number variation (CNV) data of each sample was derived from customed 139-, 168-, 425- and 520-genes panel with in-house script normalizing the sequencing depth and coverage. This study utilized the SCLC subtype heterogeneity signature gene set (*n* = 1300) as described in previous report [26]. To standardize the data, row-wise z-score normalization was applied to log2-transformed TPM (transcripts per million) expression profiles. Subsequently, hierarchical clustering analysis was performed using the pheatmap package (v1.0.12) in R to visualize and characterize the expression patterns of the gene set. The differentially expressed genes (DEGs) were identified with DESeq2 and the DEGs cluster was performed by R package pheatmap (version 1.0.12). The KEGG pathway enrichment was performed with cluster Profiler [27]. GSEA analysis was performed following the original research [28]. The immune microenvironment status and immune infiltration were calculated with the method stated in https://icbi-lab.github.io/immunedeconv/ website [29].

### Treatment, efficacy evaluation and definition of treatment group

We retrospectively counted subsequent treatment regimens after transformation. Because of the retrospective analysis, the treatment regimens were not completely consistent. Of the 23 cases of T-SCLC in this study, 9 patients treated with chemotherapy received a platinum doublet regimen, 9 patients treated with EGFR-TKI combined with chemotherapy, 2 patients with immunotherapy combined with chemotherapy, 2 patients treated with bevacizumab plus chemotherapy, and the other 1 patient continued to receive osimertinib and could not tolerate chemotherapy due to advanced age. The clinical staging of tumours in this study was performed based on the 8th edition TNM staging system. The treatment regimens were divided into matched group and unmatched group according to the molecular subtypes. Tumour response was evaluated according to Response Evaluation Criteria in Solid Tumours (RECIST) version 1.1 [30].

### Follow-up and statistical analysis

PFS was defined as the time from starting the drug treatment to confirmation of disease progression or death. Post-T-SCLC overall survival (pOS) was calculated from the date of confirmed T-SCLC to death or the last follow-up evaluation, and the objective response rate (ORR) was defined as the proportion of complete response (CR) or partial response (PR). The last follow-up time was December 31, 2022.

Chi-Square test or the Fisher’s exact test was employed to calculate the significant difference in categorical variables between two groups. Analysis of survival difference was performed using the Kaplan-Meier method and log-rank test. R software (version 3.6.1), Linux sever equipped with Python (version 2.7.13) or GraphPad Prism software (version 8.0) were employed to analyse data and carry out visualization. Statistical significance was defined as *P* < 0.05 for paired two-tail student t tests.

## Results

### Clinicopathological features of patients and study design

The enrolled patients had a diagnosis age range of 24 to 75 years, with a median age of 56.5. All patients had locally advanced or advanced-stage lung cancer at the time of diagnosis. In our cohort of EGFR-mutant LUAD patients who developed resistance to EGFR-TKI therapy by undergoing histological transformation to SCLC (23 cases), the majority harbored EGFR mutations of type 19del (17/23; 73.9%), while a minority possessed L858R mutations (6/23;26.1%). The majority were nonsmokers in the LUAD and T-SCLC groups with a percentage of 83.3% (15/18) and 78.3% (18/23), respectively. However, in the SCLC group, the majority of enrolled patients (13/20, 65%) were heavy smokers. At the time of enrolment, the ECOG performance status (PS) score indicated a compromised condition, with the score ≥ 2 points overserved in one LUAD case, three T-SCLC cases, and one SCLC case. Comprehensive characteristics of the patients are summarized in Table S1.

To accurately distinguish LUAD, SCLC, and T-SCLC, we performed whole transcriptome sequencing on patient-derived samples. We analyzed a total of 61 patients with adequate biological samples for RNA sequencing across three groups: 18 LUAD, 23 T-SCLC, and 20 SCLC. Furthermore, we included 5 paired tumor samples that were sequenced pre- and post- SCLC transformation. Additionally, these retrospectively collected tissue samples from TKI-resistant LUAD patients, T-SCLC patients, and SCLC patients for RNA sequencing, which allow us to discern expression pattern between the T-SCLC group and the other groups, thereby enhancing our understanding of their distinct molecular profiles.

We performed an unsupervised cluster analysis to determine the molecular subtype of T-SCLC and demonstrate two subtypes of T-SCLC. Through a multi-omics approach, encompassing genomic, transcriptomic, and proteomic analyses, we revealed distinct characteristics between the two subtypes. Survival analysis was then conducted, stratified by these subtypes (Graphical Abstract).

### Differential analysis of transcriptomics between LUAD and T-SCLC

**Fig. 1:**
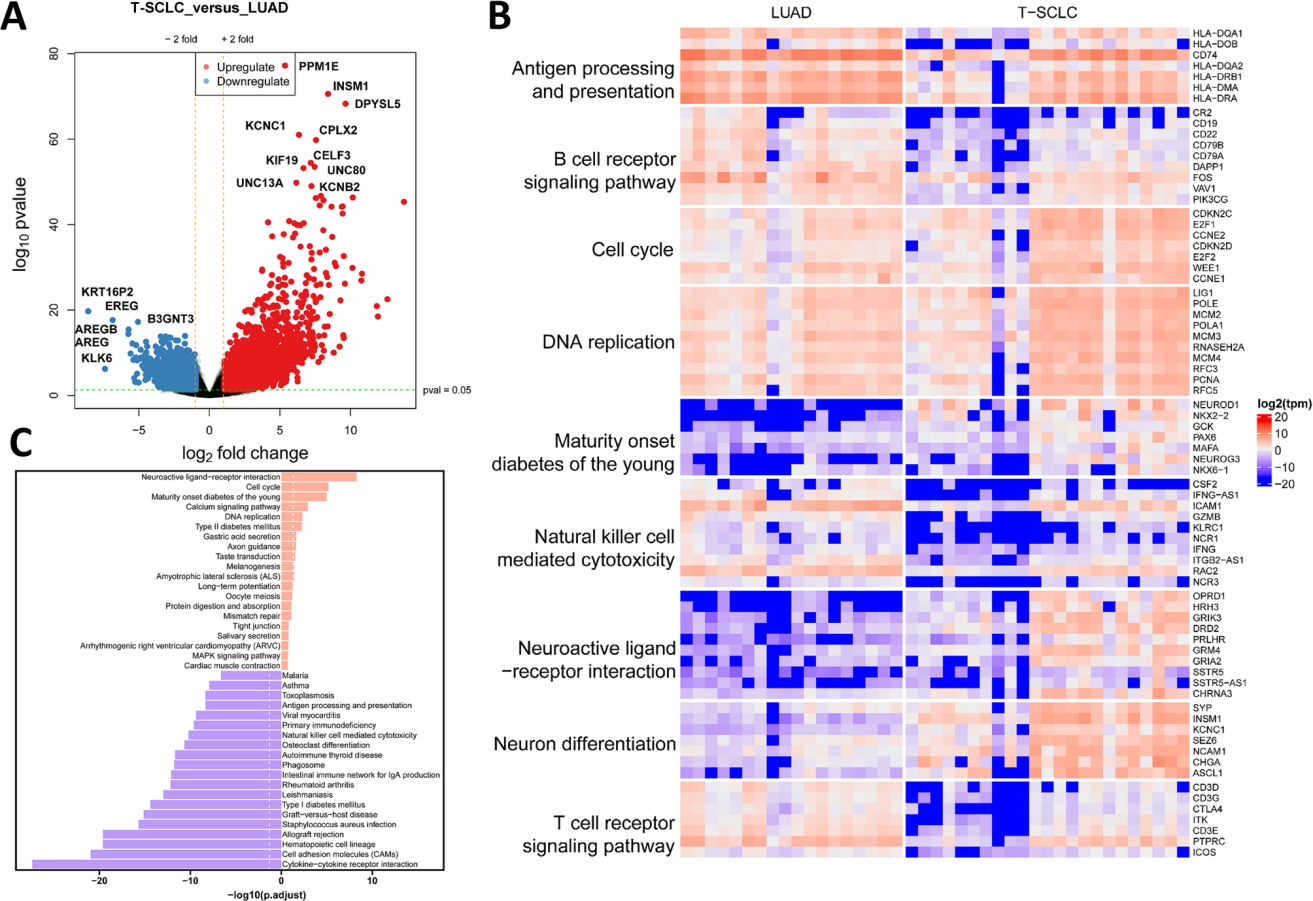
Transcriptomic differential analysis of LUAD and T-SCLC. (**A**) Volcano plot of differentially expressed genes between LUAD and T-SCLC. (**B**) Heatmap plot of differentially expressed genes between LUAD and T-SCLC. (**C**) Kyoto Encyclopedia of Genes and Genomes (KEGG) analysis of up- and down-regulated expression of genes involved in biological processes

A comprehensive differential transcriptomic analysis revealed a total of 7499 differentially expressed genes (DEGs) were between LUAD and T-SCLC samples. In comparison to LUAD, T-SCLC exhibited 4899 significantly upregulated genes and 2600 downregulated genes (Fig. 1A). The pathway enrichment analyses, particularly on the upregulated DEGs in T-SCLC (Fig. 1A and B), highlighted genes such as PPM1E, INSM1, DPYSL5, KCNC1, CPLX2, and others. As expected, NE markers were significantly upregulated in T-SCLC samples such as INSM1, CHGA, and ASCL1. In particular, INSM1 has been confirmed to be related to the pathological features of SCLC in recent studies [31, 32]. The upregulated genes are predominantly involved in processes like cell cycle (including E2F, CCNE1, WEE1), neuron differentiation (including ASCL1, INSM1, SEZ6, and CHGA), DNA replication (including MCM2, POLE, RFC), and Neuroactive ligand receptor interaction (NLRI, including GRIK3, GRM4). Conversely, downregulated genes in T-SCLC included KRT16P2, EREG, B3GNT3, which are mainly enriched in immune-related pathways, such as T/B cell receptor signalling pathway the antigen processing and presentation pathway (Fig. 1B and C). It is worth noting that the samples in the T-SCLC group exhibited discernible variations in the expression patterns of specific pathways, indicating that a high degree of tumor heterogeneity within the T-SCLC itself. This heterogeneity underscores the complexity of T-SCLC and suggests that a more nuanced understanding of its molecular landscape is essential for advancing therapeutic strategies.

**Fig. 2:**
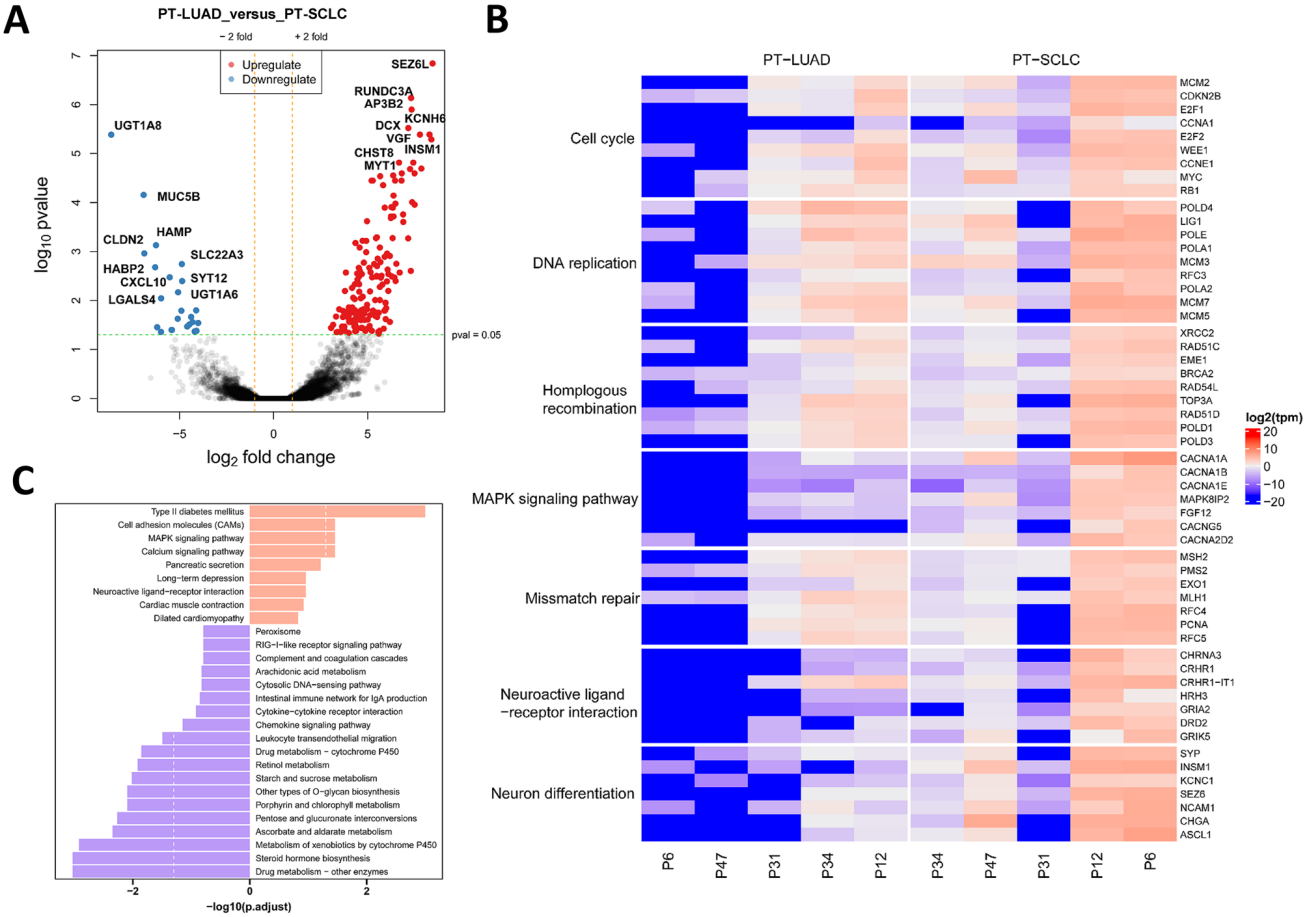
Transcriptomic differential analysis of PT-LUAD and PT-SCLC. (**A**) Volcano plot of differentially expressed genes between PT-LUAD and PT-SCLC. (**B**) Heatmap plot of differentially expressed genes between PT-LUAD and PT-SCLC. (**C**) KEGG analysis of up- and down-regulated expression of genes involved in biological processes

Given the relatively low incidence of T-SCLC, pinpointing its onset with precision remains a challenge. Our study was limited to just 5 paired samples prior to the transformation. We only had 5 paired samples available before the transformation. A comparison of pre-transformation LUAD (T-LUAD; *n* = 5) and T-SCLC (*n* = 23) samples revealed 1022 significantly upregulated genes and 436 significantly downregulated genes in the T-SCLC group. Notably, INSM1 was identified as one of the genes with significant upregulation (Figure S1A, S1B). The KEGG and gene set enrichment analysis (GSEA) showed that the pathways related to the cell cycle and homologous recombination were significantly up-regulated (Figure S1C, S1D). There was a substantial divergence in the expression patterns between T-LUAD and T-SCLC, corroborating previous results. To delve deeper into the transcriptional shifts that occur during SCLC transformation, we conducted DEG and GSEA analysis on five paired samples. These samples included paired transformed LUAD (PT-LUAD) and paired transformed SCLC (PT-SCLC). We found 190 differentially expressed genes, predominantly up-regulated genes such as SEZ6L, INSM1, RUNDC3A and others. Notably, neuroendocrine cancer-related markers were significantly upregulated after SCLC transformation, such as SYP, CHGA, INSM1, etc. (Fig. 2A). The gene heatmap starkly contrasts the expression profiles of samples before and after SCLC transformation. The heatmap also delineates the intragroup heterogeneity observed in 5 cases of paired transformed SCLC (PT-SCLC) samples (Fig. 2B). T-SCLC exhibited enhanced proliferative capacity and the MAPK pathway was significantly upregulated in T-SCLC samples, consistent with clinical observations. The enriched pathways of downregulated genes were predominantly associated with metabolism (Fig. 2C). In summary, when juxtaposed with PT-LUAD, PT-SCLC displayed a marked enrichment of neuroendocrine-related markers, hinting at a possible correlation with the activation of cell cycle pathways.

### Exploring the commonalities and distinctions between T-SCLC and SCLC

**Fig. 3:**
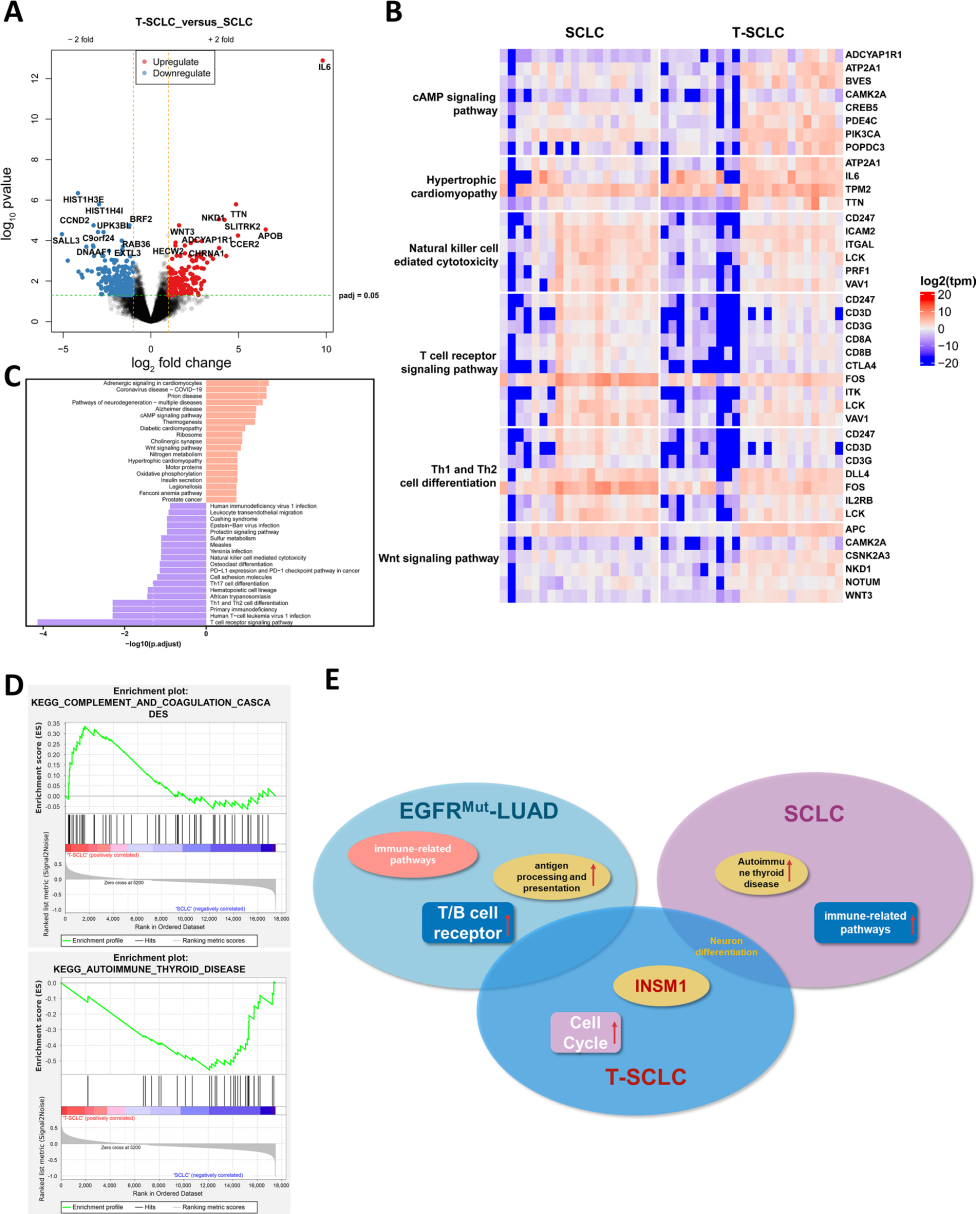
Similarities and differences between T-SCLC and SCLC. (**A**) Volcano plot of differentially expressed genes between SCLC and T-SCLC. (**B**) Heatmap plot of differentially expressed genes between SCLC and T-SCLC. (**C**) KEGG analysis of upregulated and downregulated expression of genes involved in biological processes. (**D**) Differential pathway summary for GSEA. (**E**) Summary of the differences between LUAD, T-SCLC, and SCLC

There were clear features of neuroendocrine differentiation in both T-SCLC and SCLC. Previous studies have predominantly posited that the T-SCLC shares a striking resemblance in biological characteristics with classic SCLC, but our results show that there are certain discrepancies between the two subtypes. To delineate the distinctions between the two groups of samples, we analysed the transcriptome data and identified 499 differentially expressed genes, of which 221 were up-regulated and 278 down-regulated in T-SCLC. The primary divergences are evident in the volcano plot, predominantly characterized by the downregulation of genes such as HIST1H3E and BRF2 (Fig. 3A). A comparison of the transcriptomes between T-SCLC and primary SCLC showed that highly expressed genes are associated to the Wnt signaling pathway, encompassing WNT3, NKD1, and others. While some T-SCLCs exhibited expression profiles congruent with those of SCLCs, others present pronounced divergences. Furthermore, we also discovered that T-SCLC can be stratified into two distinct clusters, each displaying considerable heterogeneity (Fig. 3B).

The GSEA and KEGG analysis has uncovered that the differentially expressed genes in both groups were primarily enriched in pathways associated with autoimmune thyroid disease and complement and coagulation cascades. These results hint at potentially heightened immune-related response in primary SCLC compared with T-SCLC (Fig. 3C and D). To elucidate the mechanisms underpinning SCLC transformation, we examined the transcriptomics of *EGFR*-mutant and transformed adenocarcinomas (T-LUAD). We identified 1051 differentially expressed genes, with 767 genes upregulated in the T-LUAD group. (Figure S2A). Upregulated differentially expressed genes by KEGG analysis were enriched for metabolic-related pathways (Figure S2B). In the LUAD group, we observed high expression of immune-related pathways (T cell receptor signaling pathway/Intestinal immune network for IgA production) according to GSEA analysis (Figure S2C). Collectively, the results above indicate a notable distinction between the groups (Fig. 3E). Both the stroma score and immune score were significantly higher in T-LUAD and LUAD compared to T-SCLC and SCLC (Figure S3A, S3B). RNA sequencing revealed that the neuroendocrine markers such as NEUROD1, NCAM1, ASCL1, CHGA, INSM1, SYP were either absent or weakly expressed in LUAD and T-LUAD, whereas these transcription factors were robustly expressed in both T-SCLC and SCLC (Figure S3C).

### Unveiling distinct molecular subtypes of T-SCLC through hierarchical clustering analysis

**Fig. 4:**
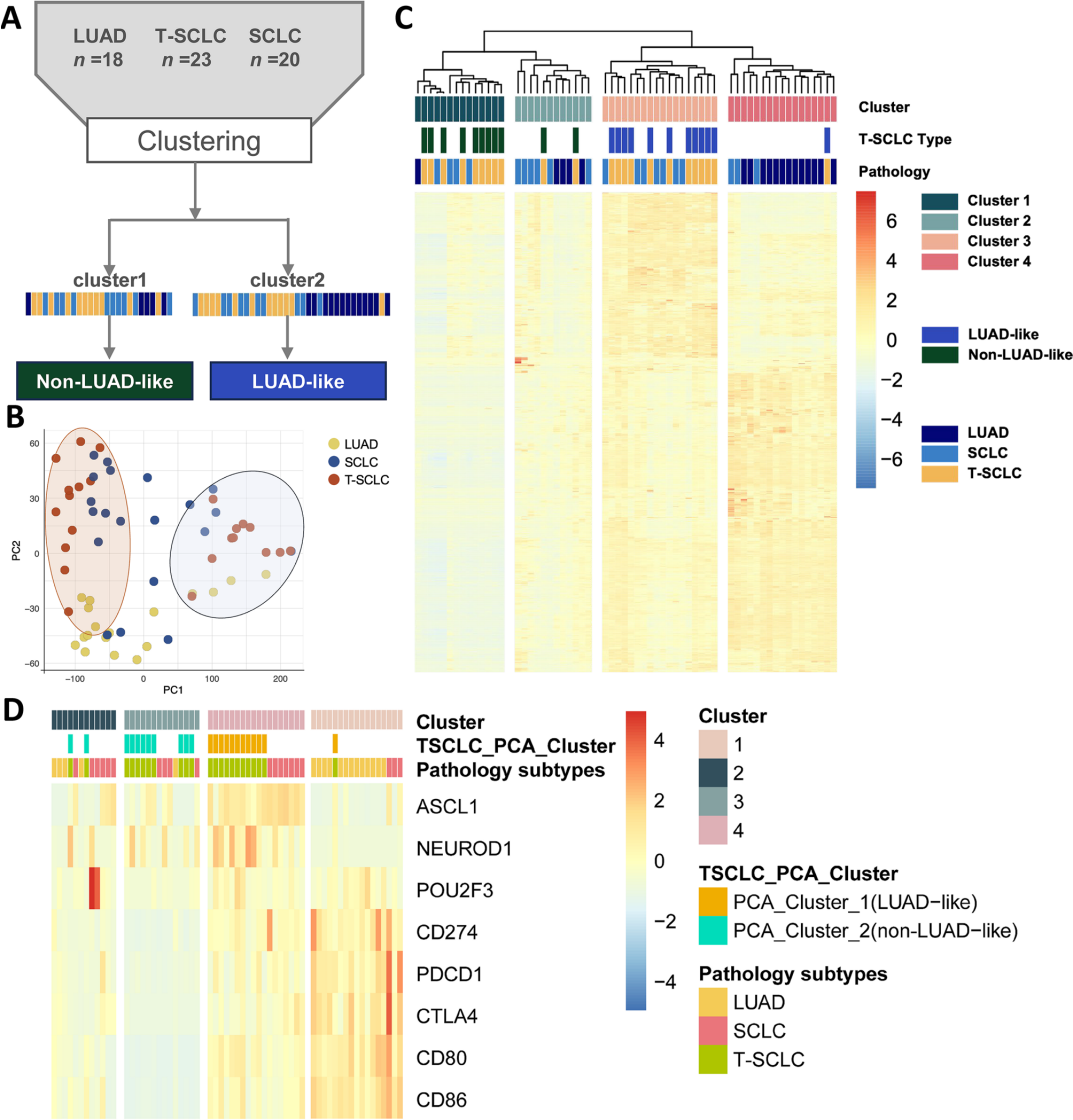
Gene expression studies on transformed small cell lung cancer subtypes. (**A**) A schematic description of the unsupervised consensus clustering. (**B**) Principal component analysis (PCA) clustering of T-SCLC subtypes. (**C**) Hierarchical clustering heatmap is shown based on gene expression, with red and blue indicating high and low expression, respectively. (**D**) Cophenetic correlation coefficients represent clustering results

The aforementioned findings demonstrate the presence of inherent heterogeneity among T-SCLC samples. To delineate the intrinsic heterogeneity of T-SCLC, we performed RNA sequencing on 61 clinical specimens (23 T-SCLC, 18 LUAD, 20 SCLC) (Fig. 4A). Unsupervised principal component analysis (PCA) demonstrated clear segregation of T-SCLC into two distinct subgroups (Fig. 4B). The robustness of this classification was further validated by hierarchical clustering analysis using 1,300 variably expressed genes (Fig. 4C).

Consistent with the PCA results, hierarchical clustering subdivided T-SCLC cases into two molecular subtypes. Unsupervised hierarchical clustering revealed distinct transcriptional bifurcation within T-SCLC specimens. Specifically, the LUAD-like subgroup (cluster 2, *n* = 12) exhibited significant transcriptional congruence with conventional lung adenocarcinomas, whereas the Non-LUAD-like subgroup (cluster 1, *n* = 11) demonstrated closer affinity to classic SCLC signatures while maintaining unique molecular features (Fig. 4A and C). In brief, the classification of T-SCLC using PCA results was consistent with hierarchical clustering, demonstrating the validity of defining two subtypes of T-SCLC (LUAD-like and Non-LUAD-like). To characterize distinct subpopulations and identify subtype-specific signature gene expression matrices, we performed functional annotation of subgroup-discriminative genes. The results demonstrated differential expression patterns of ASCL1, NEUROD1, POU2F3, and immune-related markers between LUAD-like and Non-LUAD-like subtypes; however, these features exhibited insufficient discriminative power to robustly characterize subgroup identities. However, these subsets both recapitulate and suggest heterogeneity within T-SCLC and SCLC features (Fig. 4D).

### Integrative characterization of LUAD-like and Non-LUAD-like subtypes with multi-omics

**Fig. 5:**
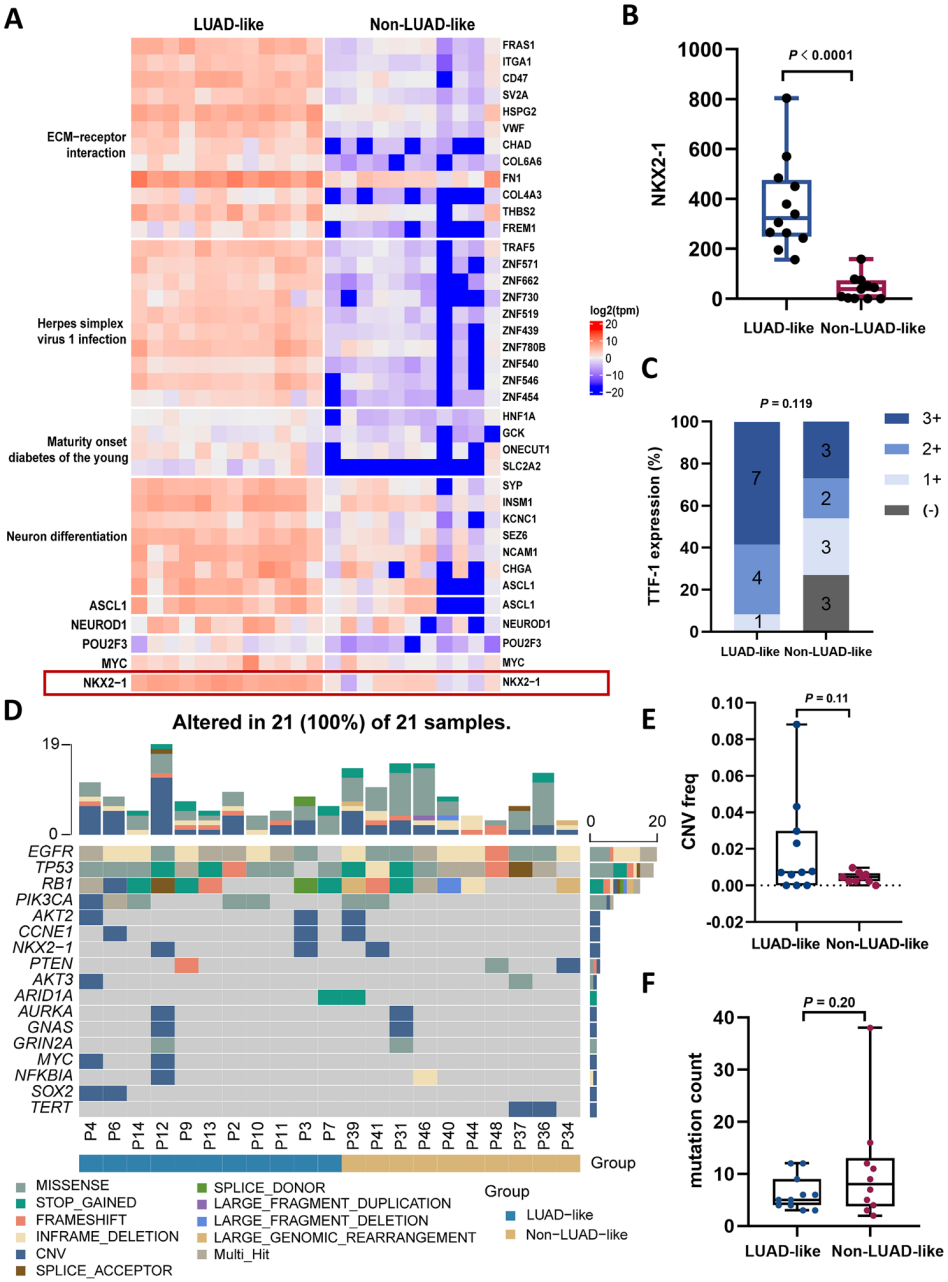
Multi-omics analysis of between LUAD-like and Non-LUAD-like subtypes. (**A**) Gene expression profiles of whole transcriptome sequencing in the LUAD-like and Non-LUAD-like group. (**B**) Transcriptome expression levels of NKX-1 in the LUAD-like and Non-LUAD-like group. (**C**) Protein expression of TTF-1 in the LUAD-like and Non-LUAD-like group. (**D**) Genomic alteration profiles of patients with LUAD-like and Non-LUAD-like groups. (**E/F**)Abbreviations CNV and TMB variants in patients with LUAD-like and Non-LUAD-like groups

Next, we conducted genomics, transcriptomics, and proteomics analyses to further elucidate the intrinsic property between the two molecular subtypes. At the transcriptomic level, these findings showed an upregulation of genes associated with ECM-receptor interaction, herpes simplex virus 1 infection, and neuron differentiation (Fig. 5A). It is particularly noteworthy that the transcription factor NKX2-1 exhibited a substantial upregulation in the LUAD-like group (mean 371.8 vs. 41.8, *P* < 0.0001) (Fig. 5B). At the protein level, TTF-1 IHC scores were elevated in the LUAD sample group but not statistically different due to sample size (*P* = 0.119) (Figure S4A, Fig. 5C). The observed disparity between the LUAD-like and Non-LUAD-like subgroups was not attributed to the presence of mixed pathological component during SCLC transformation, as the proportion of such cases was comparable between the two groups (33% vs. 36%, *P* = 0.65) (Figure S4B).

Twenty-one tissue samples also have undergone the panel-based DNA sequencing to study the genomic underpinnings of T-SCLC transformation. It was found that almost all T-SCLC retained the original EGFR mutation (20/21, 95.2%). The frequency of EGFR mutations differs between LUAD-like and Non-LUAD-like samples before- and after transformation, but no discernible patterns emerged (Figure S4C). The mutational landscape shows that the top 4 genes with the highest mutation frequency in T-SCLC were EGFR, TP53, RB1 and PIK3CA (Fig. 5D). Intriguingly, the distribution of EGFR, TP53 and RB1 mutation sites were observed to be analogous between LUAD-like and Non-LUAD-like groups (Figure S4D). At the DNA level, the mean number of CNVs was numerically higher in the LUAD-like group (mean 0.02 vs. 0.00, *P* = 0.11) (Fig. 5E), while the mutation count was numerically more frequent in the Non-LUAD-like group (mean 6.27 vs. 10.70, *P* = 0.20), neither of which showed a statistical significance (Fig. 5F).

### Survival analysis of matched and unmatched groups defined according to T-SCLC subtypes

**Fig. 6:**
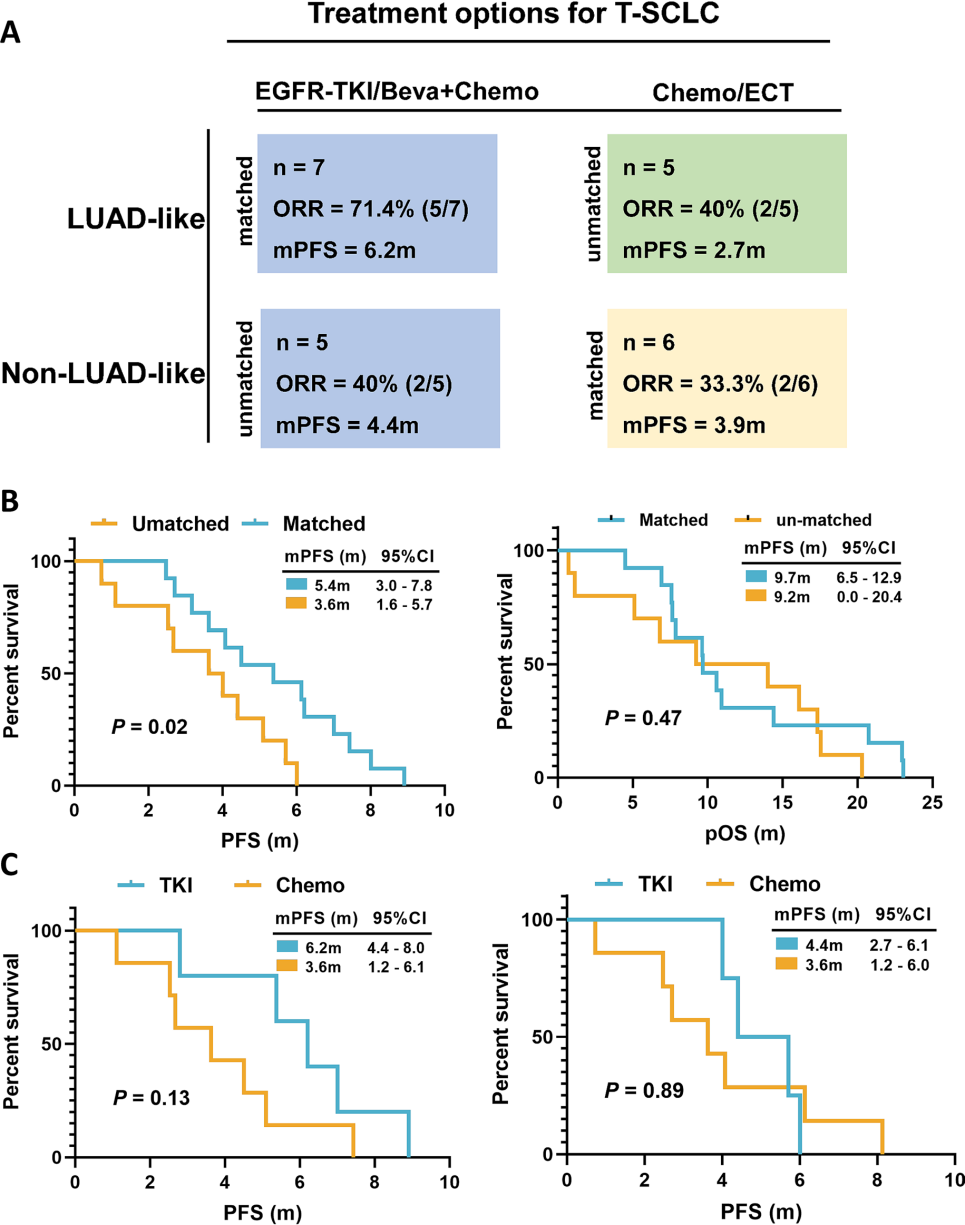
Survival analysis of matched and unmatched groups defined according to T-SCLC subtype. (**A**) Treatment options for T-SCLC were categorized into matched and unmatched groups, delineated by the alignment of specific therapies with corresponding pathologies. (**B**) Evaluation of progression-free survival (PFS), Post-T-SCLC overall survival (pOS) for matched and unmatched groups. (**C**) PFS presentation of subgroups was based on whether TKI was used or not

The treatment modalities were divided into matched and unmatched groups, based on the alignment between specific therapies and corresponding pathologies. The Matched Group was defined as T-SCLC patients receiving subtype-specific therapies: LUAD-like subtypes treated with EGFR-TKI or Bevacizumab based therapy, or Non-LUAD-like subtypes treated with platinum-based chemotherapy ± immunotherapy. The Unmatched Group comprised patients whose post-transformation therapies did not align with these molecular subtype-guided regimens. This classification was based on whether therapeutic strategies corresponded precisely to the identified pathological subtypes. The matched group consists of 13 cases, including patients with LUAD-like subtypes who received adenocarcinoma-targeted treatments (EGFR-TKI/ Bevacizumab plus chemotherapy), and those with Non-LUAD-like subtypes who underwent SCLC-specific therapy (Chemotherapy for SCLC) (Table S2). The unmatched group consists of 10 cases. Among the LUAD-like cases, T-SCLC patients treated with EGFR-TKI/bevacizumab plus chemotherapy showed a higher response (ORR 71.4%, mPFS 6.2 months) compared to those who received other treatments (ORR 40%, mPFS 2.7 months) (Fig. 6A). The matched group had a significantly extended median PFS compared to the unmatched group (5.4 months vs. 3.6 months, *P* = 0.02). However, there was no significant difference in overall survival (OS) between the matched and unmatched groups for T-SCLC (9.7 months vs. 9.2 months, *P* = 0.47) (Fig. 6B). In the LUAD-like group, continuing EGFR-TKI treatment showed a trend towards longer PFS compared to discontinuation (6.2 months (95%CI, 4.4–8.0 months) vs. 3.6 months (95%CI, 1.2–6.1 months), *p* = 0.13). Conversely, the continued use of EGFR-TKI in the Non-LUAD-like group did not yield any clinical benefit (4.4 months vs. 3.6 months, *P* = 0.89) (Fig. 6C).

## Discussion

Lineage plasticity is considered a crucial factor in the failure of EGFR-TKI treatment. However, there is controversy surrounding the origin of tumor cells and no effective therapeutics are available to target lineage conversion [33]. When SCLC transformation occurs, the tumor becomes highly malignant, invasive, prone to metastasis, and has a poor prognosis. In this study, we aim to clarify the distinctions between T-SCLC, LUAD, and primary SCLC through multi-omics analysis. Additionally, we summarize the molecular characteristics of T-SCLC. To our knowledge, this is the first comprehensive molecular analysis conducted on T-SCLC using transcriptome sequencing to identify two distinct molecular subtypes: LUAD-like and Non-LUAD-like. Previous research by us and others has shown that most T-SCLCs retain their original *EGFR* mutation when transforming from *EGFR*-mutated adenocarcinomas during resistance to EGFR-TKIs [15, 34]. These T-SCLCs harbor an *EGFR* mutation but are insensitive to EGFR-TKIs. Furthermore, our transcriptome analysis reveals significant differences in differentially expressed genes and enriched pathways after SCLC transformation compared to LUAD. Notably, we observed a significant upregulation of INSM1 as a transcription factor following SCLC transformation. INSM1 serves as a specific marker for lung neuroendocrine carcinoma with diagnostic value equivalent to neuroendocrine histochemical indicators Synaptophysin (Syn), CD56 (NCAM), and Chromogranin A (CGA) [35, 36]. There is evidence suggesting that INSM1 plays an important role in regulating neuroendocrine differentiation [37, 38]. The relationship between INSM1, EGFR, and cell cycle has been confirmed to be a key factor in NE transformation of prostate cancer [38, 39]. Future work should include preclinical models to confirm the role of INSM1 in SCLC transformation. Previous studies have shown that both T-SCLC and de novo SCLC respond well to platinum-based chemotherapy [15, 40]. However, our analysis results indicate that certain T-SCLC profiles share similarities with SCLC, while others exhibit significant differences. In summary, T-SCLC has a unique molecular signature that sets it apart not only from LUAD but also from classical SCLC.

In general, classic SCLC exhibits typical neuroendocrine signature and is thought to originate primarily from pulmonary neuroendocrine cells. De novo SCLC has been divided into 4 proposed subtypes based on differentially expressed genes based on transcriptomics, which is helpful for developing personalized therapy [22, 41]. Memorial Sloan Kettering Cancer Center (MSKCC) studied a cohort of mixed histology LUAD/SCLC to mimic T-SCLC, and they found that T-SCLC can also be defined as these 4 subtypes [24]. Both published studies and our results suggest that T-SCLC is not a homogeneous tumour. In this study hierarchical clustering analysis identified 2 distinct molecular subtypes within T-SCLC, namely LUAD-like and Non-LUAD-like. In order to rule out the difference between the two subtypes caused by mixed pathological components in the initial diagnosis of transformed SCLC, our results show that there is no significant difference in the proportion of mixed pathological types between LUAD-like and Non-LUAD-like (33% vs. 36%, *P* = 0.65).Therefore, the differences between the two subgroups are not due to mixed pathological types but to their own differences. The LUAD-like subgroup had significantly higher expression of NKX2-1 compared to Non-LUAD-like. Subtype could be determined by transcriptomics and proteomics, TTF-1 (NKX2-1) is a potential representative marker of LUAD-like. Interestingly, the LUAD-like subgroup was more prone to adenocarcinoma but had higher expression of neuroendocrine-related markers compared with Non-LUAD-like as shown in Fig. 5A. Thus, T-SCLC is a specific pathological and genetic phenotype, distinct from adenocarcinoma and classical SCLC [42]. TTF-1 and ASCL1 cooperatively promote neuroendocrine differentiation and tumor growth. Conversely, high MYC expression suppresses both ASCL1 and TTF-1, driving SCLC toward a poorly differentiated variant phenotype. Molecular subtyping based on the expression patterns of TTF-1, ASCL1, and MYC may help predict treatment response and guide personalized therapeutic strategies for SCLC.

Next, let’s discuss our first proposal to develop a treatment plan based on molecular subtyping. Treatment options are minimal when undergoing SCLC transformation; hence, further treatments need to be evaluated. The matched group was defined as two scenarios, one with EGFR-TKI or bevacizumab in combination with chemotherapy for the LUAD-like subgroup and the other with chemotherapy ± immunotherapy for the Non-LUAD-like subgroup. If not, this patient was categorised into the unmatched group. The results of this study showed that the PFS of the matched group was significantly prolonged. There is currently no standard treatment for T-SCLC, although research into treatment options for T-SCLC has continued in recent years [43]. In conclusion, the selection of individualised treatment regimens for T-SCLC based on molecular typing is necessary.

Regarding the limitation of our study, although multi-omics analyses have been performed to characterize T-SCLC, and this is the first time that the treatment of T-SCLC has been proposed according to molecular subtype, but it is undeniable that certain limitations still exist. First, selection bias may occur due to the retrospective, small sample, single-centre nature. Second, the matched samples before and after inclusion were insufficient due to the low incidence of T-SCLC and the inability to accurately identify SCLC transformation. Then, the needle biopsy sample may not necessarily reflect the full picture of the tumor’s histological characteristics. Last, the results of this study still lack in vitro model validation, but our team has successfully constructed a T-SCLC organoid model to explore the T-SCLC treatment model.

In summary, T-SCLC is molecularly distinct from both LUAD and classical SCLC. It can be further classified into two subtypes: LUAD-like and Non-LUAD-like. Tailoring therapeutic protocols based on these subtypes could help identify the most suitable treatment for T-SCLC.

## Electronic supplementary material

Below is the link to the electronic supplementary material.

Supplementary Material 1: Figure S1. Expression profiling for T-LUAD and T-SCLC. (A) Volcano plot of differentially expressed genes between two groups. (B) Heatmap plot of differentially expressed genes between two groups. (C) KEGG analysis of up- and down-regulated expression of genes involved in biological processes. (D) Up-regulated differential gene pathway summary for GSEA

Supplementary Material 2: Figure S2. Expression profiling for T-LUAD and LUAD. (A) Volcano plot of differentially expressed genes between T-LUAD and LUAD. (B) Differential pathway summary for GSEA. (C) KEGG analysis of up- and down-regulated expression of genes involved in biological processes

Supplementary Material 3: Figure S3. Intergroup differential expression of immune microenvironment and neuroendocrine cancer markers. (A) Stroma score between groups. (B) Immune score between groups. (C) Expression values for key neuroendocrine differentiation markers are plotted for each group as boxplots

Supplementary Material 4: Figure S4. Multi-omics differential analysis in LUAD-like and Non-LUAD-like. (A) TTF-1 immunohistochemical (IHC) staining results in T-SCLC cases. (B) Percentage of LUAD-like and Non-LUAD-like mixed pathology types. (C) Changes in maximum mutational abundance of EGFR before and after transformation between the two subgroups. (D) Differences in the distribution of key gene loci between the two subgroups

Supplementary Material 5: Table S1. Clinical characteristics of patients for three groups

Supplementary Material 6: Table S2. The treatment approaches of T-SCLC for matched and unmatched groups

## Data Availability

No datasets were generated or analysed during the current study.

## Declarations

### Lead contact

Further information and requests for resources and reagents should be directed to and will be fulfilled by the lead contact, Jin-Ji Yang (yangjinji@gdph.org.cn).

## Abbreviations

SCLC: Small-cell lung cancer
EGFR: Epidermal growth factor receptor
T-SCLC: Transformed small-cell lung cancer
LUAD: Lung adenocarcinoma
TKI: Tyrosine kinase inhibitor
NMF: Non-negative matrix factorization
NSCLC: Non-small cell lung cancer
EP: Etoposide/platinum
mPFS: Median progression-free survival
RNA-seq: RNA sequencing
IHC: Immunohistochemistry
HE: Hematoxylin and eosin
FFPE: Formalin-fixed paraffin-embedded
NGS: Next-generation sequencing
ARMS: Amplification refractory mutation system
SNVs: Single nucleotide variants
SNVs: Single nucleotide variants
DEGs: Differentially expressed genes
KEGG: Kyoto Encyclopedia of Genes and Genomes
GSEA: Gene set enrichment analysis
pOS: Post-T-SCLC overall survival
ORR: Objective response rate
CR: Complete response
PR: Partial response
PS: The physical status
T-LUAD: Transformed- lung adenocarcinoma
PT-LUAD: Paired transformed LUAD
PT-SCLC: Paired transformed SCLC
